# Supplementary material for: The AP-1 Transcription Factor Fosl-2 Regulates Autophagy in Cardiac Fibroblasts during Myocardial Fibrogenesis
Source: Int J Mol Sci. 2021 Feb 13;22(4):1861. doi: 10.3390/ijms22041861 (PMC7917643; doi:10.3390/ijms22041861)
Supplement: Supplementary file 1 [file ijms-22-01861-s001.pdf]

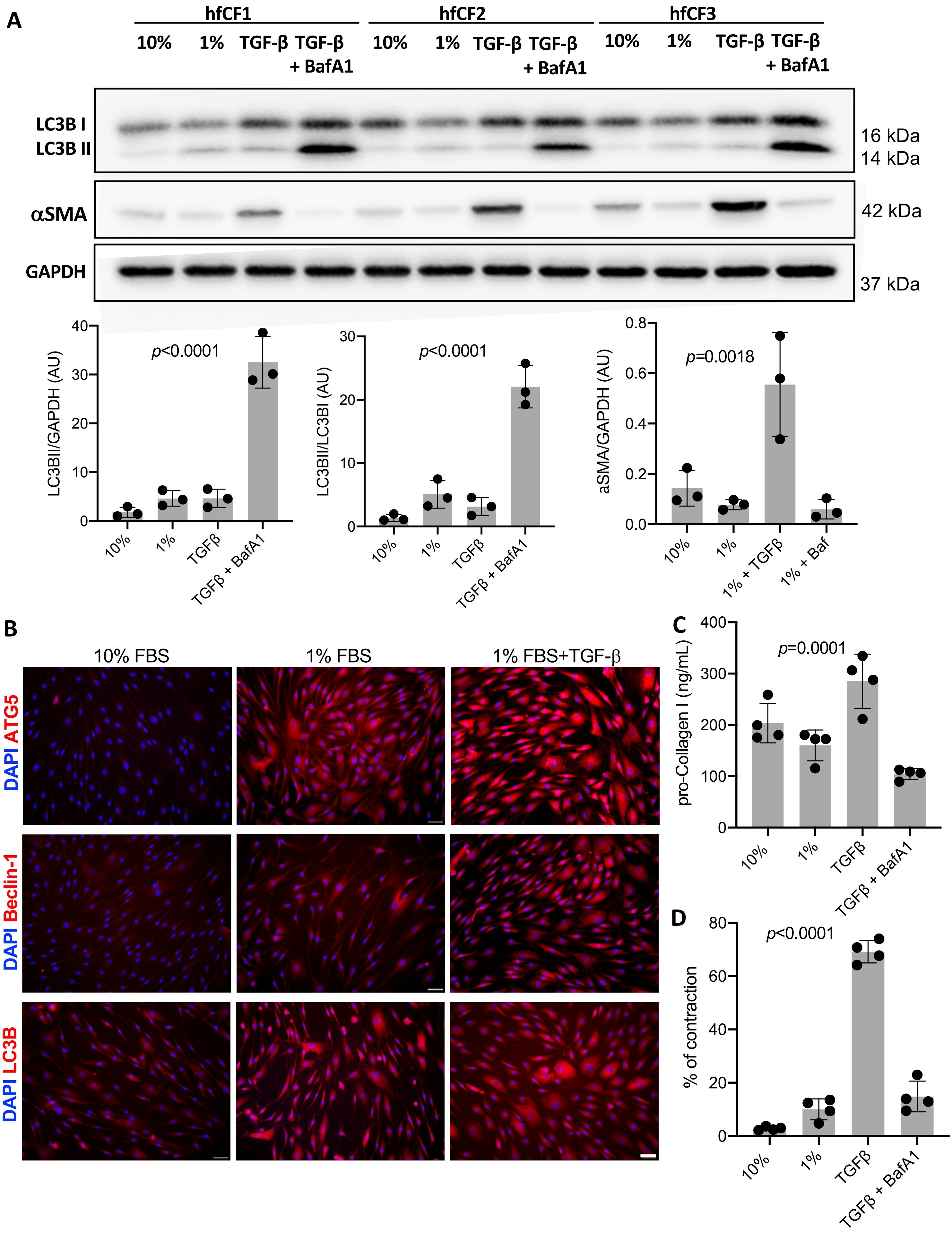

Supplementary Figure 1. Activation of autophagy process in human foetal cardiac fibroblasts (hfCFs).
A) HfCFs were cultured in 10% or 1% FBS supplemented medium, stimulated with or without TGF-β for 24h and preincubated with BafA1 for 4h, where indicated. Representative immunoblots and corresponding densitometry analysis of LC3B and αSMA (one-way ANOVA, n=3). **B**) Representative pictures of ATG5, Beclin-1 and LC3B immunofluorescence staining (n=3). Nuclei are stained with DAPI (blue). Scale bars: 10 µm. **C**) Pro-collagen I concentration in supernatants collected from hfCFs (one-way ANOVA, n=4). **D**) Quantification of gel contraction 96h after seeding hfCFs in collagen gel (one-way ANOVA, n=4).

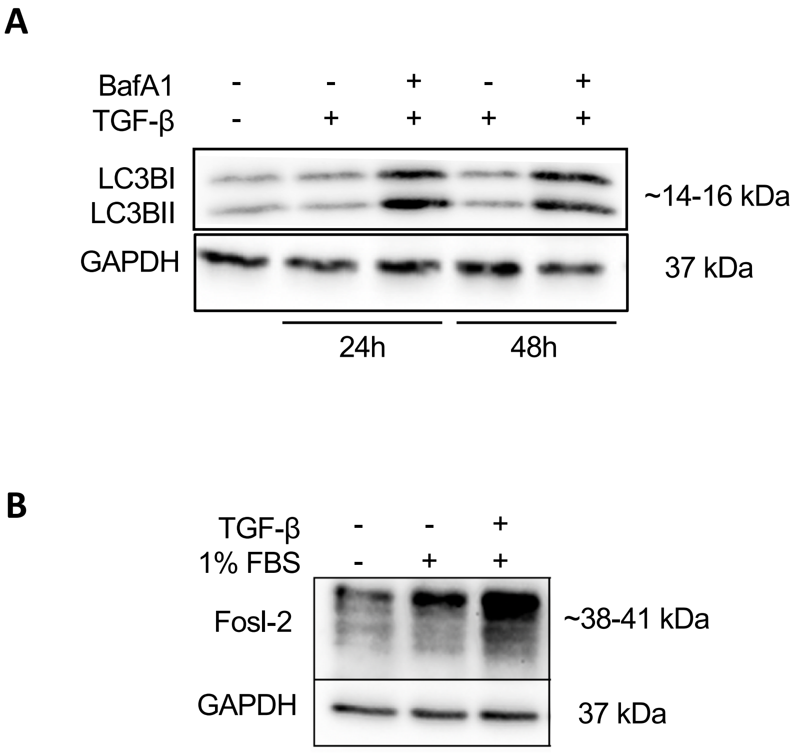

Supplementary Figure 2. Co-activation of autophagy and TGF-β signalling in mouse Fosl-2^{wt} cardiac fibroblasts. A) Cardiac fibroblasts were treated with 50 nM BafA1 for 4h, and stimulated with TGF-β for 24h or 48h. Immunoblots show LC3B and GAPDH. **B) C**ardiac fibroblasts were cultivated in 10% or 1% FBS supplemented medium and stimulated with or without TGF-β for 24h (n=3). Immunoblots show Fosl-2 and GAPDH.

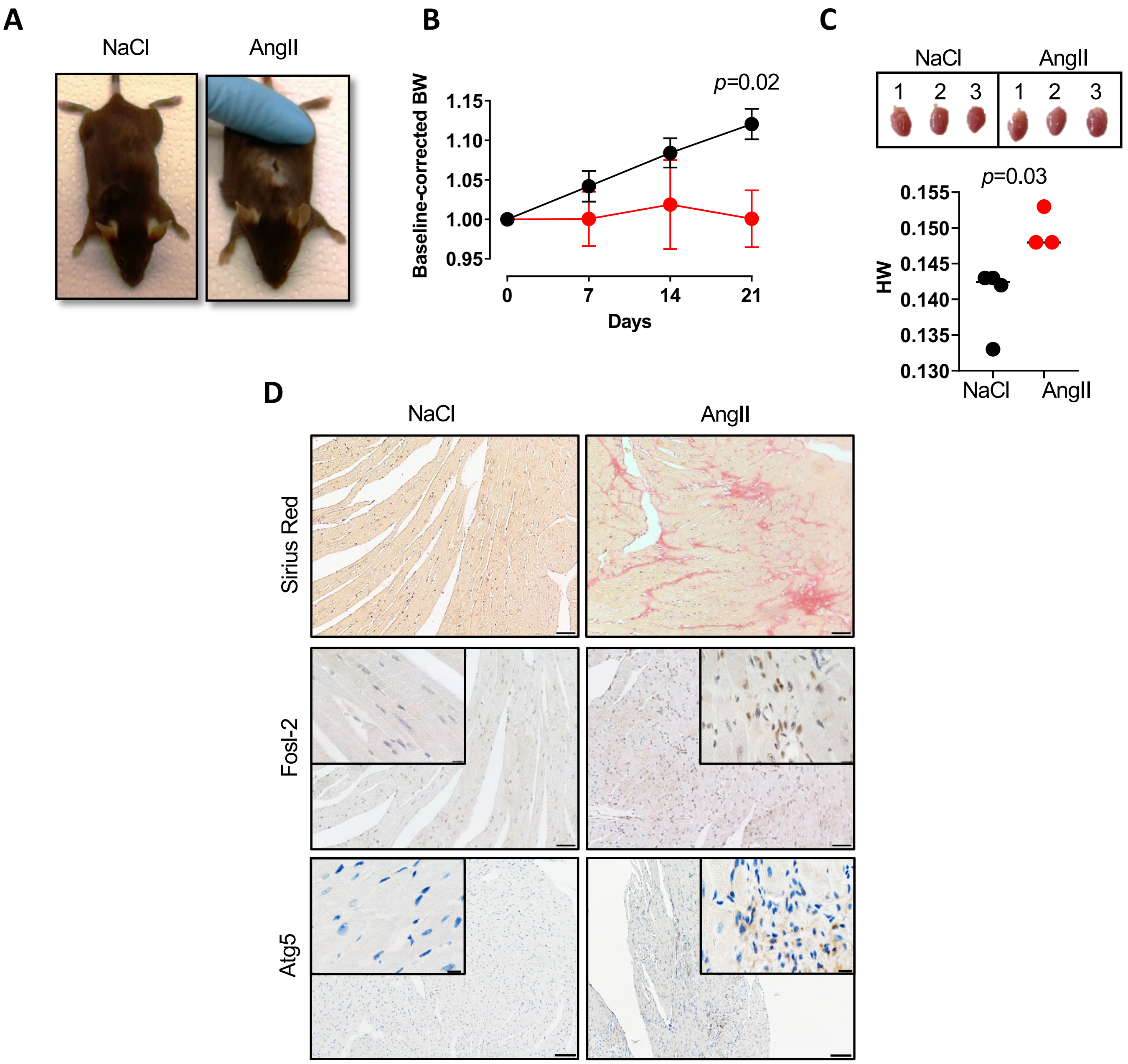

Supplementary Figure 3. Angiotensin (Ang) II-induced cardiac hypertrophy model. C57BL/6 mice were implanted with saline solution (NaCl) or angiotensin II filled osmotic minipumps for 3 weeks. **A)** The examples of skin lesion at the second week of treatment. **B)** Body weights (BW) of Ang II-treated mice (red) and NaCl control mice (black) over 3-week treatment (n=3-4, Mann-Whitney *U* test). **C)** Representative pictures of hearts and heart weights (HW) measured at the endpoint of the experiment (n=3-4, Mann-Whitney *U* test). **D)** Representative pictures of Sirius Red staining, Fosl-2 and Atg5 IHC staining on heart sections. Scale bar: 50 µm, insert scale bar: 10 µm.

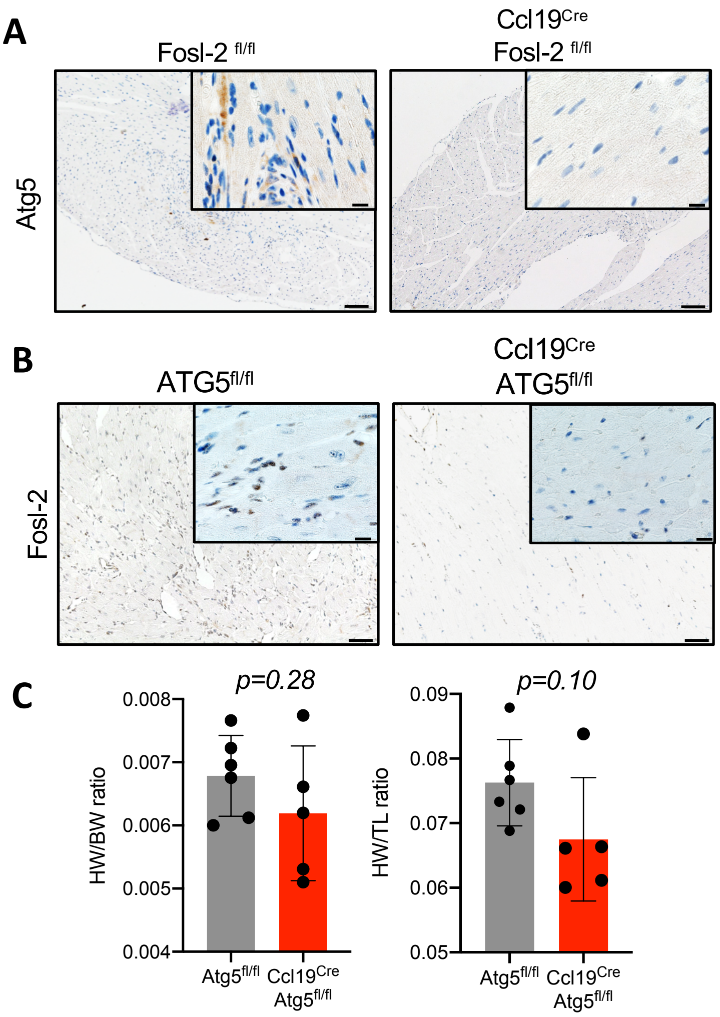

Supplementary Figure 4. Characteristic of Ccl19^{Cre}Atg5$^{fl/fl}$ and Ccl19^{Cre}Fosl-2$^{fl/fl}$ mice in cardiac hypertrophy model. Mice were implanted with angiotensin II filled osmotic minipumps and analysed 3 weeks later. **A)** Representative pictures of Atg5 IHC staining of myocardial sections from Fosl-2$^{fl/fl}$ control mice and Ccl19^{Cre}Fosl-2$^{fl/fl}$ mice. Scale bar for A, C: 100 µm, scale bars in inserts: 10 µm, (n=6). **B)** Representative pictures of Fosl-2 IHC staining of myocardial sections from Atgf5$^{fl/fl}$ control mice and Ccl19^{Cre}Atg5$^{fl/fl}$ mice (n=5-6). **C)** Heart weight (HW)/body weight (BW) and HW/tibial length (TL) ratios of Atgf5$^{fl/fl}$ control mice and Ccl19^{Cre}Atg5$^{fl/fl}$ mice (n=5-6, Mann-Whitney *U* test).

Supplementary material

Conflicts of Interest

The disclosure statement of Prof. O. Distler:

<u>Disclosure Statement of Potential Conflicts of Interest</u>

Scleroderma associated Disclosures (3 years backwards, 2017-01/2020):
Prof. Dr. O. Distler had consultancy relationship and/or has received research funding from:
Abbvie, Actelion, Acceleron Pharma, Amgen, AnaMar, Baecon Discovery, Blade Therapeutics, Bayer, Boehringer Ingelheim, Catenion, Competitive Drug Development International Ltd , CSL Behring, ChemomAb, Curzion Pharmaceuticals, Ergonex, Galapagos NV, Glenmark Pharmaceuticals, GSK, Inventiva, Italfarmaco, iQone, iQvia, Lilly, medac, Medscape, Mitsubishi Tanabe Pharma, MSD, Novartis, Pfizer, Roche, Sanofi, Target Bio Science and UCB in the area of potential treatments of scleroderma and its complications.
In addition, Prof. Distler has a patent mir-29 for the treatment of systemic sclerosis issued (US8247389, EP2331143).

Grants from Actelion, Bayer, Boehringer Ingelheim, Mitsubishi Tanabe
Patent issued: mir-29 for the treatment of systemic sclerosis (US8247389, EP2331143).
Comment: To investigate potential treatments of scleroderma and its complications

---

Detailed Disclosures (3 years backwards)

- **Speaker fee on Scleroderma and related complications**: Actelion, Bayer, Boehringer Ingelheim, Medscape, Novartis, Roche
- **Speaker fee on rheumatology topic other than Scleroderma**: Menarini, Mepha, MSD, iQone, Novartis, Pfizer, Roche
- **Consultancy fee for Scleroderma and its complications**: Abbvie, Actelion, Acceleron Pharma, Amgen, AnaMar, Bayer, Baecon Discovery, Blade Therapeutics, Boehringer, CSL Behring, ChemomAb, Curzion Pharmaceuticals, Ergonex, Galapagos NV, GSK, Glenmark Pharmaceuticals, Inventiva, Italfarmaco, iQvia, medac, Medscape, Mitsubishi Tanabe Pharma, MSD, Roche, Sanofi, UCB
- **Consultancy fee for rheumatology topic other than Scleroderma:** Abbvie, Amgen, Lilly, Target BioScience, Pfizer
- **Project scoring fee for Abbvie Rheumatology Grant**: Abbvie
- **Interview fee on Scleroderma and related complications**: Catenion
- **Research Grants to investigate the pathophysiology and potential treatment of Scleroderma and its complications**: Actelion, Bayer, Boehringer Ingelheim, Competitive Drug Development International Ltd, Mitsubishi Tanabe
